# The Effect of Pore Structure on Impact Behavior of Concrete Hollow Brick, Autoclaved Aerated Concrete and Foamed Concrete

**DOI:** 10.3390/ma15124075

**Published:** 2022-06-08

**Authors:** Jian Liu, Yuzhe Ren, Rui Chen, Yuedong Wu, Weidong Lei

**Affiliations:** 1 Key Laboratory of Ministry of Education for Geomechanics and Embankment Engineering, Hohai University, Nanjing 210098, China; 20170053@hhu.edu.cn (J.L.); yuzherenyuzhe@outlook.com (Y.R.); 2 Geotechnical Engineering Research Center of Jiangsu Province, Nanjing 210098, China; 3 Engineering Research Center of Dredging Technology of Ministry of Education, Hohai University, Changzhou 213000, China; 4 Department of Civil and Environmental Engineering, Harbin Institute of Technology, Shenzhen 518055, China; cechenrui@hit.edu.cn (R.C.); wdlei@hitsz.edu.cn (W.L.)

**Keywords:** drop-weight impact test, pore structure, concrete hollow brick (CHB), autoclaved aerated concrete (AAC), foamed concrete (FC), energy absorption

## Abstract

Porous concrete is an energy absorption material, which has been widely used in civil engineering, traffic engineering and disaster reduction engineering. However, the effect of pore structure on the impact behavior of the porous concrete is lacked. In this study, a series of drop-weight impact tests were carried out on three typical types of porous concrete, i.e., concrete hollow brick (CHB), autoclaved aerated concrete (AAC) and foamed concrete (FC), to investigate the effect of pore structures on their impact behavior. For comparison, static load tests were also conducted as references. According to the damage to the samples, the developments of impact force, strain, contact stress–strain relationship and absorbed energy during drop-weight during the impact test were measured and analyzed. The results show that the ratio between the peak impact stress and compressive strength of CHB was 0.44, while that of AAC and FC increased to about 0.6, indicating that the small and uniform pore structure in AAC and FC had a higher resistance against impact load than the hollow cavity of CHB. In addition, the elastic recovery strain in AAC increased by about 0.2% and its strain at peak contact stress increased by about 160% for a comparison of CHB, implying that a small open pore structure could enhance ductility. Besides, the peak contact stress of FC was close to that of AAC during impact loading, while the strain at peak contact stress of FC increased by about 36% compared with AAC, revealing that the closed-pore structure could further enhance the deformation potential. Correspondingly, the energy absorption rates of CHB, AAC and FC were 85.9 kJ/s, 54.4 kJ/s and 49.7 kJ/s, respectively, where AAC decreased by about 58% compared with CHB, and FC decreased by about 10% compared with AAC.

## 1. Introduction

Porous concrete, as an energy absorption material, has been widely used for impact resistance in civil buildings [1], traffic engineering [2,3] and disaster reduction engineering [4]. To assess the ability of the materials to absorb the energy resulting from impact, a drop-weight impact test and split Hopkinson pressure bar test are commonly employed. Even though split Hopkinson pressure bar tests could provide a higher and well-controlled strain rate, a drop-weight impact test has been wild-used for its easy operation. Ranade et al. adopted drop-weight impact tests and found that the strain-hardening of concrete promotes energy absorption [5]. Furthermore, Li found that porous concrete has a better impact resistance than normal concrete [6]. A maximum energy absorption was observed when the density of porous concrete was 1000 kg/m^3^ and its porosity ranged from 50 to 60% [7]. However, its porosity was not clarified as being either open pore or closed pore. At a fixed porosity, low drop heights permitted the materials to undergo a degree of elastic recovery, whereas sample size had only a marginal influence on energy absorption on impact [8]. Li et al. carried out split Hopkinson pressure bar tests to investigate the effect of size and shape on concrete impact behavior and found that the size effect is unimportant while shape effect plays a significant role and increases with the strain rate [9]. Bai et al. also conducted split Hopkinson pressure bar tests on porous concrete and revealed an exponential relationship between total energy absorption and incident energy change rate [10]. However, there are many types of porous concrete, among which concrete hollow brick (CHB), autoclaved aerated concrete (AAC) and foamed concrete (FC) are typical types of porous concrete available on the civilian market. The difference regarding the impact behavior between these three types of porous concrete is not clear. In addition, the effect of pore structure on the impact behavior of porous concrete has not been studied.

CHB commonly has one or more hollow cavities and its sidewalls are made by a slurry of cement, water, sand and gravel [11]. Different from CHB, AAC is produced by aluminum powder, quartz sand, calcined gypsum, lime and/or cement and water [12]. It is noteworthy that since it does not contain coarse aggregate, it has the advantage of low density [13]. When reaching a temperature of 190 °C and a pressure of 8 to 12 bar, quartz sand reacts with calcium hydroxide to form calcium silicate hydrate, whereas aluminum powder reacts with calcium hydroxide and water to form hydrogen gas. The hydrogen gas created air bubbles of up to about 3 mm in diameter. Compared with CHB, the pores in AAC are small and uniform. For FC, a slurry mixed by foaming agents, cement, sand and water is used [14]. The foaming agent is used to produce air bubbles before mixing, where sodium dodecyl sulfate, cetyltrimethyl ammonium bromide, hydrolyzed protein are commonly used [15]. As demonstrated by microscopic observation, the pore size of AAC and FC ranges only from 1 to 5 mm [16]. As compared with AAC, most pores of FC are closed [17,18]. It is expected that pore structure would largely affect the impact behavior of porous concrete. However, the comparison of impact behavior among the three typical types of porous concrete is limited and desired.

In this study, drop-weight impact tests were carried out on the three typical types of porous concrete with similar porosity and static compressive strength to investigate the effect of pore structure, i.e., hollow cavity for CHB, a small and uniform open pore structure for AAC and a small and uniform closed-pore structure for FC. The developments of impact force, strain and contact stress–strain relationship and absorbed energy were analyzed. In addition, static load tests were conducted to provide a reference for comparison. The effect of closed-pore structure on impact behavior and energy absorption are also highlighted.

## 2. Laboratory Experiments

### 2.1. Sample Size

Considering the marginal influence of sample size [8] and the porous concrete size available on the civilian market, CHBs with dimensions of 190 mm × 190 mm× 190 mm were used. Each CHB has one rectangle enclosure with 25 mm in thickness and its void volume fraction accounts for 54.3%. In contrast to the CHB samples, cube samples of 100 mm × 100 mm × 100 mm were used for AAC and FC cut from masonry blocks of 400 mm × 200 mm × 100 mm. These samples described above were used for both static load tests and drop-weight tests.

### 2.2. Porosity

According to the RILEM method CPC 11.3 [19], the open pore content (or accessible porosity) was measured using the vacuum saturation method for each sample. The sample was firstly oven-dried to 105~110 °C until its mass reached a steady value. The drying process typically lasted 24 h [20]. Then, the sample was immersed in water and vacuumed (negative pressure of 0.750 mHg) for up to 24 h. Finally, the open pore content was determined as shown in Equation (1):

(1) $$n_{0}=\frac{V-\Delta V}{V}$$

where *n*_0_ is open pore content of the sample, *V* is the volume of the sample and Δ*V* is the increase in the water volume in the container for immersing the sample under vacuum.

The total porosity could be calculated through the mass of the fine granules crushed by porous concrete. Similar to the method for measuring open pore content, the sample was firstly oven-dried to 105~110 °C until its mass reached a steady value. Subsequently, the sample was crushed into fine granules with a diameter of 1 mm, which is smaller than the pore size of the porous concrete [16]. Finally, the fine granules were immersed in water and vacuumed for up to 24 h. The total porosity could be determined as shown in Equation (2):

(2) $$n_{t}=\frac{V-\Delta V_{t}}{V}$$

where *n_t_* is total porosity of the sample, Δ*V_t_* is the increase in the water volume in the container for immersing the fine granules under vacuum. It was assumed that the total porosity of the sample consists of open pores and closed pores. Then, the closed-pore content was calculated as shown in Equation (3):

(3) $$n_{c}=n_{t}-n_{0}$$

where *n_c_* is closed-pore content of the sample. Table 1 lists the properties of pores for the three types of porous concrete. Total porosities ranging from 58.9% to 63.2% were chosen to obtain the density of maximum energy absorption (i.e., 1000 kg/m^3^) reported by Jones and Zheng [7]. It is noteworthy that six samples of each type of porous concrete were chosen to measure the total porosity and closed-cell pore content, whereas Table 1 only shows the arithmetic mean of the measured data. In order to avoid the effect of total porosity on the impact behavior of porous concrete, three types of concrete with a similar total porosity (60 ± 3.5%) were chosen, and the standard deviation of the porosity of CHB, AAC and FC are 1.57%, 1.17% and 1.73%, respectively. Compared with CHB, the pores of AAC and FC were small and uniform. The main difference between AAC and FC was the closed-cell pore content.

### 2.3. Static Load Test

The static load test was carried out using a compression testing machine (DNS 300, Changchun Sinotest Equipment Co., Ltd., Changchun, China) for comparison with the results of the drop-weight tests. For each sample, the strain rate for the test was 0.05% per second and the corresponding compressive load-deformation curve was recorded for analysis. Two samples of each type of porous concrete were chosen for the static load test. Table 1 also lists the average compressive strengths for the static load tests of the three types of porous concrete, where they shared similar compressive strengths (3.75 ± 0.25 MPa).

### 2.4. Drop-Weight Impact Test

**Figure 1:**
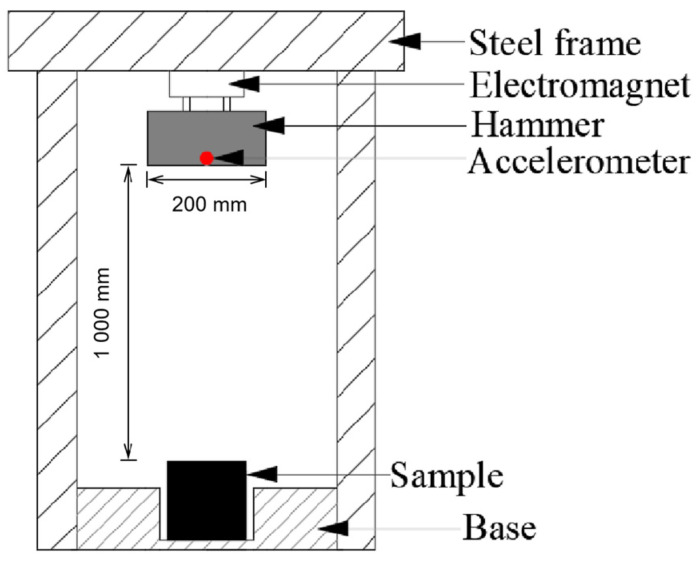
Setup for drop-weight test.

Figure 1 shows the setup for the drop-weight impact test used in this study. It consists of a steel frame and a 20 kg drop hammer of with a 200 mm × 200 mm bottom surface. The hammer was mounted under the frame with an electromagnet that could be turned on and off easily and rapidly. Once the electromagnet was turned on, the hammer stuck to the frames. After the electromagnet was turned off, the hammer began to drop under self-weight. Each sample was set 1000 mm vertically below the hammer and the drop energy for each test was set as 200 J.

For each test, an accelerometer was installed in the middle, close to the bottom of the hammer, to measure the acceleration of the hammer during the drop-weight test. The accelerometer could be sampled at a sampling frequency of 100 kHz, whereas its maximum recordable acceleration was ±5000 m/s^2^. During the drop-weight test, the accelerometer was connected to a data logger with frequency of up to 1 MHz.

Before each drop-weight test, the accelerometer was calibrated first. Then, the accelerometer was fixed into the hammer and the hammer was mounted under the frame by turning on the electromagnet. Vertically, below the hammer, the sample was placed in the base. Finally, the electromagnet was turned off and the acceleration data were recorded. For each porous concrete, five samples were tested. Their average value was then considered for analysis in this study.

## 3. Results

### 3.1. Damage to Samples

**Figure 2:**
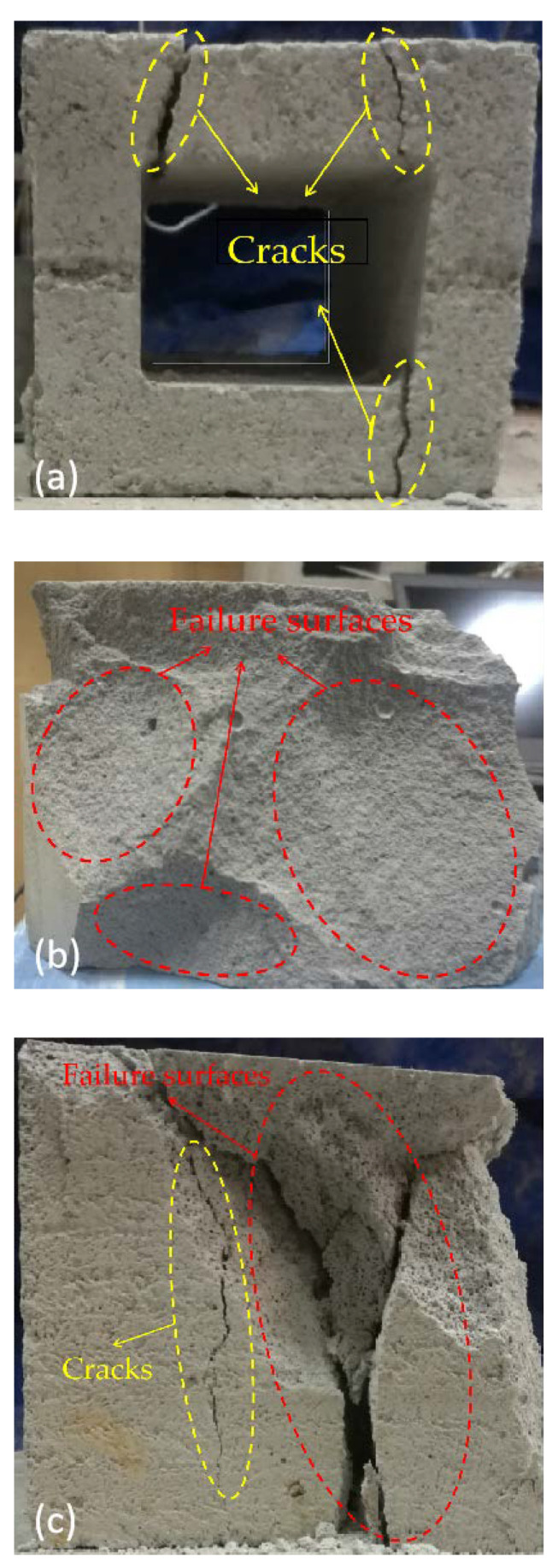
Samples after drop-weight test: (**a**) CHB; (**b**) AAC; (**c**) FC.

Figure 2 shows photos of the damaged CHB, AAC and FC after drop-weight tests. For CHB, local failure was observed and three cracks mainly occurred at the corner due to the hole-edge stress concentration, in which two cracks were positioned at the top and one crack was observed at the bottom as shown in Figure 2a. At the top, the two cracks with about a 5 mm width were inclined approximately 20° towards the hollow cavity. This is similar to the baroclinic damage in the concrete beam under static load, indicating that the high oblique tensile stresses occurred at the corner. At the bottom, the crack ran parallel to the vertical side. This may be due to the bending moment caused by the oblique tensile stresses at the top. Even though there were three cracks in CHB, no spalling was observed under the impact loading, indicating that CHB has good integrity.

In contrast to CHB, AAC and FC collapsed with a loss of integrity due to the occurrence of failure surfaces throughout the sample as shown in Figure 2b,c, respectively. Firstly, many continuous cracks propagated in an unstable manner. As these cracks developed, failure surfaces could be observed and then samples were fractured with debris spalling. It should be noted that the degree of damage to the AAC and FC samples was larger than that for CHB. This indicates that greater deformation occurred in the samples of AAC and FC under the impact load. In addition, the pores of the upper part of the sample at about 5 mm in contact with the drop hammer were clearly compacted, while the pores further from the impact of the drop hammer experienced no significant change, indicating that the failure of AAC and FC were progressive.

### 3.2. Impact Force

**Figure 3:**
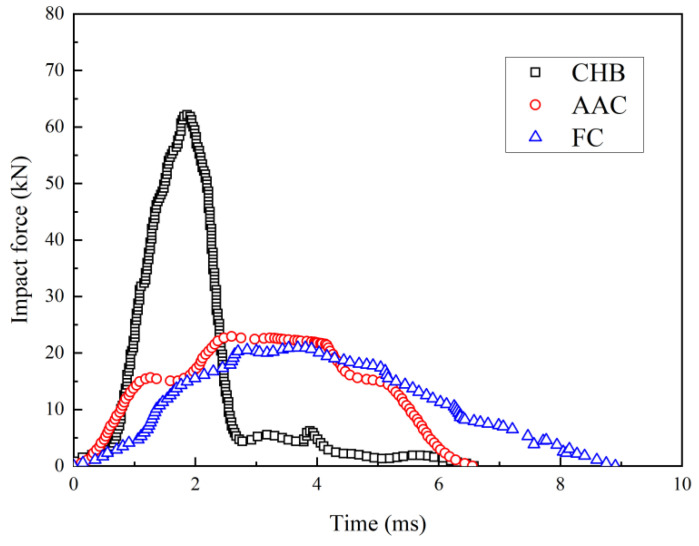
Evolution of impact force with time during drop-weight tests.

Figure 3 shows the development of the impact force for CHB, AAC and FC during drop-weight tests. The time when the hammer began to contact the sample was taken to be zero. The impact force is derived by the acceleration of the hammer as shown in Equation (4):

(4) F=(a−g)m

where *F* is impact force, *m* is the mass of the hammer, *g* is gravitational acceleration and *a* is the acceleration measured by the accelerometer in the hammer. For simplicity, the upward direction was taken as the positive direction and thus *g* was negative. As time elapsed, the developments of impact force for the three types of porous concrete were similar, where the impact force increased first and after reaching a peak value, began to decrease. The peak impact force of CHB was 62.1 kN, while it was approximately 22.9 kN and 20.9 kN for AAC and FC, respectively, i.e., approximately 66% smaller than that for CHB. The peak impact force occurred at about 1.8 ms for CHB, but at about 3 ms for AAC and FC. The time for the peak impact force for AAC and FC was about 60% longer than that for CHB, indicating better ductility for AAC and FC. Based on the momentum-impulse equilibrium, the longer the impact time, the smaller the impact force from the hammer.

At the post-peak region, the impact force of CHB decreased to about 5 kN in about 1 ms and then decreased slowly to zero, as shown in Figure 3. This can be attributed to the pore structure of CHB. As mentioned previously, CHB has a hollow cavity and the failure model could be simplified as a concrete beam. The bearing capacity was diminished after the failure of the top edge, as shown in Figure 2a. For a similar porosity (i.e., 60 ± 3%), the impact force at the post-peak region for AAC and FC was larger than that for CHB. This occurred due to the presence of many small and uniform pores in AAC and FC, which acted similarly to a sponge or cushion. After the peak value, even though some cracks or failure surfaces occurred (see Figure 2b,c) other pores without failure were still able to undergo the impact load, prolonging the contact time. It should be noted that the impact force decreased to zero at about 6.5 ms for AAC, which was about 2.5 ms earlier than that for FC. In other words, the decrease rate of the impact force for FC was only about 60% of that for AAC. This may be attributed to the closed-pore structure in FC. Under the impact load, the air in the pores was compressed. Because the pore was closed, the air could not be expelled rapidly and the contact time was prolonged.

### 3.3. Strain

**Figure 4:**
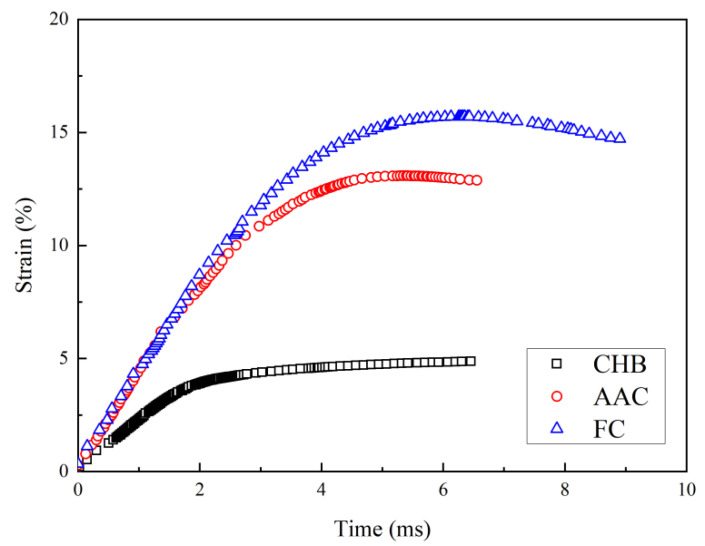
Development of strain with time during drop-weight tests.

In order to normalize sample deformation, strain was chosen in the following analysis. Figure 4 shows the strain-time histories of the three types of porous concrete during the drop-weight tests. The strain was derived by twice-integrating the measured acceleration with respect to time [21], as shown in Equation (5):

(5) $$\varepsilon =\frac{\int {}_{0}^{t_{1}}\left(v_{0}-\int {}_{0}^{t}adt\right)dt}{h}$$

where *t* is time, *ε* is the strain of the sample over the time interval [0, *t*_1_], *v*_0_ is the initial velocity of the hammer (i.e., −4.43 m/s due to the drop height of 1 m in this study), *h* is the height of the sample. For simplicity, the compression was taken as the positive value for strain. As expected, the strain of the sample increased with time due to the increase in strain energy converted from potential energy. Initially, the increase in the strain rate was 21.7 s^−1^ for CHB, while it was about 43.6 s^−1^ for AAC and FC, almost twice the level of CHB. After 1.8 ms, the increase in the strain rate of CHB began to decrease and then the strain curve converged to 4.8%, while the peak strain of AAC was 13.1%, about 2 times larger than that of CHB. Compared with AAC, the peak strain of FC was 15.7%, thereby indicating that the amount of strain that the sample underwent before failure was greater, confirming the better ductility of FC.

After peak strain, the increase in the strain rate began to decrease after 3 ms only for AAC and 4 ms for FC, respectively, indicating that a degree of elastic recovery occurred for AAC and FC. A similar elastic recovery was also observed by Ranade et al. [5]. It may derive from the residual elasticity caused by the small and uniform pores in AAC and FC. The elastic recovery strain, which is the difference between peak stain and residual strain, was 0.2% for AAC, but 1.0% for FC, confirming that the closed air bubbles in FC could better cushion against deforming.

## 4. Discussion

### 4.1. Contact Stress–strain Relationship

**Figure 5:**
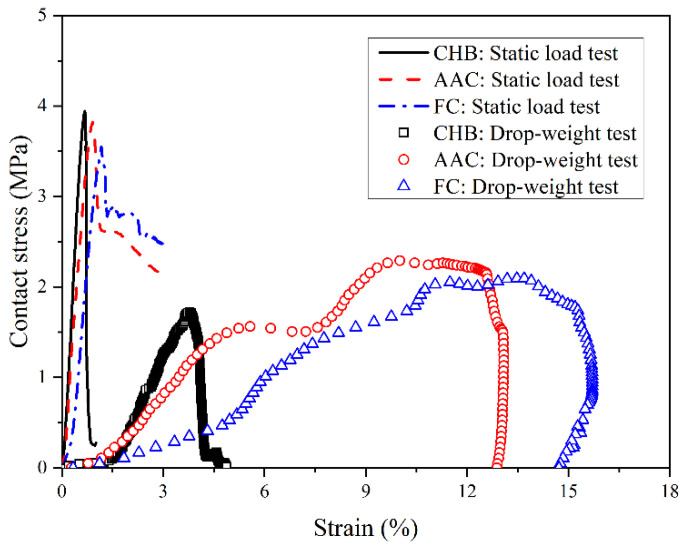
Contact stress–strain curves during static load and drop-weight tests.

Figure 5 shows the relationship between contact stress and its corresponding strain during the drop-weight tests. For comparisons, the results measured by the static load test are also included in the figure as a reference. The symbols represent experimental observations measured by the drop-weight test and the curves in the static load test. The contact stress is derived by the contact force as shown in Equation (6):

(6) $$\sigma =\frac{F}{A}$$

where *σ* is contact stress and *A* is contact area. It is worth noting that we supposed the hammer was in full contact with the sample until the sample had been damaged. For each sample of porous concrete, the contact stress–strain curves first increased linearly, with a relatively low slope until about 1.5% strain. The slopes of the contact stress–strain curves at the initial stage ranged from 5 to 8 MPa, at about 10% of the slopes in the static load test due to the slow increase in the contact stress. However, the low slope observed in this study at the initial stage was not found for concrete in previous studies [21] because porous concretes were not used in their studies. As previously analyzed, porous concretes behaved similarly to a spring. The smaller the strain, the smaller the contact stress was. At the initial contact, the contact stress was small, whereas the strain change was large because the initial velocity was maximum. After the initial stage, the contact stress for the three porous concretes increased nonlinearly until a peak value. For CHB, the peak contact stress measured in the drop-weight test was 1.7 MPa, 44.7% of that measured in the static load test. However, the strain at the peak contact stress measured by the drop-weight test was 3.7%, about 5 times of that measured in the static load test. Similar changes were also observed by the other two types of porous concrete. The peak contact stresses measured in the drop-weight test were 2.29 and 2.11 MPa for AAC and FC, respectively. Their peak contact stress was about 30% larger than that of CHB. Meanwhile, their strain at the peak contact stress measured in the drop-weight test ranged from 10% to 13.5%, about 3 times that of CHB. After the peak value, the contact stress rapidly decreased to zero with elastic recovery.

On the other hand, for the static load test, the peak stresses of the CHB, AAC and FC were 3.94, 3.77 and 3.48 MPa, respectively. The largest difference in the peak contact stress between these three types of porous concrete was smaller than 0.5 MPa. The maximum peak stress occurred for CHB, but the minimum peak stress was determined for FC. Different from the static load test, the peak contact stress was minimum for CHB and maximum for AAC in the drop-weight test. Thus, in order to avoid the influence of the compressive strength, the ratio between the peak contact stress and compressive strength was selected. The ratio for CHB was 0.44, smaller than 1. It indicates that the impact load that led to the failure of the sample was much smaller than its compressive strength. Compared with CHB, the ratios for AAC and FC were about 0.6, indicating that a small and uniform pore structure could enhance impact resistance against impact load.

Furthermore, the strains at the peak contact stress for the three types of porous concrete subjected to static load ranged from 0.67% to 1.18%. In the drop-weight test, the strain at the peak contact stress was 3.78% for CHB. Compared with CHB, the strain at the peak contact stress for AAC was 10.01%, indicating that the small and uniform pores could increase the ductility at the peak contact stress. For FC, the strain at the peak contact stress was 13.68%, implying that the closed-pore structure could further enhance the ductility at the peak contact stress.

### 4.2. Absorbed Energy of Porous Concrete

**Figure 6:**
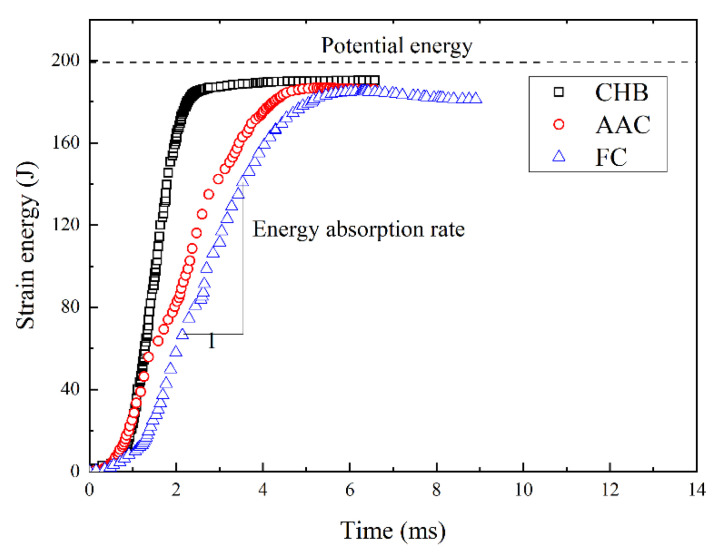
Evolution of strain energy with time during drop-weight tests.

Figure 6 presents the development of absorbed energy with time during the drop-weight tests. The energy absorbed by the sample (i.e., the work done by the hammer to the sample) is equal to the volume of the sample multiplied by the area below the contact stress–strain curve in Figure 5. For each porous concrete sample, the energy increased slowly in the first 0.8 ms. As previously analyzed, this characteristic can be attributed to the slow increase in the contact stress. Subsequently, the energy increased almost linearly to the maximum. For comparison, the potential energy was also included in the figure, calculated using Equation (7):

(7) $$E_{p}=mgH$$

where *E_p_* is potential energy, *m* is the mass of the hammer, *g* is gravitational acceleration and *H* is the drop height. The energy absorbed by each porous concrete at the end was close to the potential energy of the hammer at the start of each test, indicating that the accuracy of the measured acceleration was satisfactory. The energy conversion ratios from potential energy to strain energy for CHB, AAC and FC were 96.3%, 94.9% and 92.5%, respectively. Some energy was dissipated as sound or probably heat. It indicates that most of the energy was absorbed when physically damaging the porous concrete.

In addition, as shown in Figure 6, the energy absorption rate (i.e., the slope of the absorbed energy curve) was 54.4 kJ/s for AAC, which is about 58% smaller than the 85.9 kJ/s for CHB, which can be attributed to the increase in the impact load bearing capacity and ductility caused by the small and uniform pores in the porous concrete. For FC, the energy absorption rate was 49.7 kJ/s, 10% smaller than that for AAC, due to the increase in the deformation potential caused by the closed-pore structure in the porous concrete.

## 5. Conclusions

In this study, static load and drop-weight impact tests were carried out on three types of porous concretes with different pore structure, i.e., CHB, AAC and FC. The development of impact force, strain and absorbed energy was analyzed and discussed. Based on the experimental results, the following conclusions may be drawn:

The ratio between the peak contact stress and compressive strength was 0.44 for CHB. It indicates that the impact load causing the failure of the porous concrete was much smaller than its compressive strength. The ratio increased to about 0.6 for AAC and FC, indicating that the small and uniform pore structure could enhance impact resistance against impact load.

Compared with CHB, the elastic recovery strain in AAC increased by about 0.2%. Moreover, strain at peak contact stress was increased by about 160% for ACC, thereby indicating that the small open pore structure could enhance ductility. Correspondingly, the energy absorption rate of AAC was decreased by about 58%.

Compared with AAC, the peak contact stress of FC remained almost constant. However, the strain at the peak contact stress of FC was increased by 36%, indicating that the closed-pore structure could further enhance ductility. Correspondingly, the energy absorption rate of FC was decreased by about 10%.

In the future, the boundary effect could be considered and a large-scale falling-ball impact test could be conducted to investigate the properties of porous concrete members. Additionally, numerical simulation on the impact behavior of the different types of porous concrete could be conducted to investigate the related mechanism.

## Figures and Tables

**Table 1:** Pore structure properties of the three types of porous concrete.

	CHB	AAC	FC
Total porosity (%)	58.9	63.2	62.5
Closed-cell pore content (%)	2.6	1.4	42.1
Compressive strength (MPa)	3.94	3.77	3.48

## Data Availability

The data presented in this study are available with the article.
